# Synergistic Effect between Cryptotanshinone and Antibiotics against Clinic Methicillin and Vancomycin-Resistant *Staphylococcus aureus*

**DOI:** 10.1155/2014/450572

**Published:** 2014-03-24

**Authors:** Jeong-Dan Cha, Jeong-Ho Lee, Kyung Min Choi, Sung-Mi Choi, Jeong Hye Park

**Affiliations:** ^1^Department of Research Development, Institute of Jinan Red Ginseng, Jinan, Republic of Korea; ^2^Department of Oriental Medicine Resources, College of Environmental and Bioresource Sciences, Chonbuk National University, Republic of Korea; ^3^Department of Dental Hygiene, Daegu Health College, Daegu, Republic of Korea; ^4^Department of Nursing, Dong-eui University, Republic of Korea

## Abstract

Cryptotanshinone (CT), a major tanshinone of medicinal plant *Salvia miltiorrhiza* Bunge, demonstrated strong antibacterial activity against clinic isolated methicillin and vancomycin-resistant *Staphylococcus aureus* (MRSA and VRSA) in this experiment. The CT was determined against clinic isolated MRSA 1–16 with MIC and MBC values ranging from 4 to 32 and 8 to 128 **μ**g/mL; for MSSA 1-2 from 16 to 32 **μ**g/mL and 64 to 128 **μ**g/mL; for VRSA 1-2 from 2 to 4 **μ**g/mL and 4 to 16 **μ**g/mL, respectively. The range of MIC_50_ and MIC_90_ of CT was 0.5–8 **μ**g/mL and 4–64 **μ**g/mL, respectively. The combination effects of CT with antibiotics were synergistic (FIC index <0.5) against most of tested clinic isolated MRSA, MSSA, and VRSA except additive, MRSA 4 and 16 in oxacillin, MRSA 6, 12, and 15 in ampicillin, and MRSA 6, 11, and 15 in vancomycin (FIC index < 0.75–1.0). Furthermore, a time-kill study showed that the growth of the tested bacteria was completely attenuated after 2–6 h of treatment with the 1/2 MIC of CT, regardless of whether it was administered alone or with ampicillin, oxacillin, or vancomycin. The results suggest that CT could be employed as a natural antibacterial agent against multidrug-resistant pathogens infection.

## 1. Introduction

*Staphylococcus aureus *(*S. aureus*) is one of the most important pathogens in both hospitals and the community and can cause numerous syndromes in humans, such as furuncle, carbuncle, abscess, pneumonia, meningitis, bacterial arthritis, myocarditis, endocarditis, osteomyelitis, and septicemia [1–3]. Methicillin-resistant* Staphylococcus aureus *(MRSA) is a significant problem in hospitals and communities worldwide, and awareness of MRSA in animals and reports of its zoonotic spread have increased in recent years [3, 4]. The increasing prevalence of methicillin-resistant* Staphylococcus aureus* (MRSA) has led to widespread, increased use of vancomycin [5, 6]. Subsequently, numerous reports of elevated minimum inhibitory concentrations (MIC) to vancomycin among MRSA isolates have surfaced, concomitant with increased global vancomycin exposure to MRSA [7, 8]. Greater than 60% of* S. aureus *isolates are now resistant to methicillin (oxacillin), and some strains have developed resistance to more than 20 different antimicrobial agents; new agents are therefore needed for the treatment of* S. aureus *[9, 10]. Plant medicines are used on a worldwide scale to prevent and treat infectious diseases. They are of great demand both in the developed and developing countries for the primary health care needs due to their wide biological and medicinal activities, higher safety margin and lesser costs [11, 12]. Plants are rich in a wide variety of secondary metabolites such as tannins, alkaloids, terpenoids, and flavonoids having been found* in vitro *since they have antimicrobial properties and may serve as an alternative, effective, cheap, and safe antimicrobial for the treatment of microbial infections [12–15]. At the same time, because of the difficulty in developing chemical synthetic drugs and because of their side effects, scientists are making more efforts to search for new drugs from plant resources to combat clinical multidrug-resistant microbial infections [11, 12, 16].

The main components of* S. miltiorrhiza *can be divided into two groups: hydrophilic compounds such as salvianolic acids and lipophilic chemicals, including diterpenoid and tanshinones [17]. The second group of components, labeled as tanshinone I, tanshinone II, and cryptotanshinone, are the major bioactive constituents and have various kinds of pharmacological effects including antibacterial, antioxidant, and antitumor activities and prevention of angina pectoris and myocardial infarction [18–20]. The cryptotanshinone (CT) exhibits antimicrobial activity against a broad range of Gram-positive bacteria, including* S. aureus*, and Gram-negative bacteria as well as other microorganisms [18, 21]. The hexane and chloroform fractions of* S. miltiorrhiza *evidenced profound antimicrobial activity and inhibited resistant gene expression against* Staphylococcus aureus *and MRSA (methicillin-resistant* Staphylococcus aureus*) [21, 22].

In this study, the antimicrobial activities of cryptotanshinone (CT) against methicillin and vancomycin-resistant* Staphylococcus aureus *isolated in a clinic were assessed using broth microdilution method and the checkerboard and time-kill methods for synergistic effect of the combination with antibiotics.

## 2. Materials and Methods

### 2.1. Plant Extraction and Isolation

The air-dried roots (2.0 kg) of* S. miltiorrhiza *were crushed and extracted with MeOH (8 L × 3) at room temperature. The MeOH extracts (210 g) were evaporated and suspended in distilled water and partitioned sequentially with CH_2_Cl_2_, EtOAc, and* n*-BuOH. The CH_2_Cl_2_ soluble fraction was concentrated* in vacuo, *and its crude extract (28 g) was subjected to silica gel column chromatography using a CHCl_3_-MeOH gradient solvent system to provide ten fractions (A1–A10). Fraction A4 was further subjected to silica gel column chromatography with a CHCl_3_-MeOH (10 : 1) to yield five fractions (B1–B5). Fraction B3 was purified on recycling prep-HPLC (JAIGEL GS column and 220 nm) eluted with MeOH (4.0 mL/min) to yield cryptotanshinone (98 mg). The structure of cryptotanshinone was identified by comparing its spectral data with those published [23, 24].

### 2.2. Preparation of Bacterial Strains

16 isolates of methicillin-resistant* Staphylococcus aureus*, 2 isolates of methicillin-sensitive* S. aureus *(MSSA), and 2 isolates of vancomycin-resistant* S. aureus* (VRSA) were purchased from the Culture Collection of Antimicrobial Resistant Microbes (CCARM); standard strains of methicillin-sensitive* S. aureus *(MSSA) ATCC 25923 and methicillin-resistant* S. aureus *(MRSA) ATCC 33591 were used as well (Table 1). Antibiotic susceptibility was determined in testing the inhibition zones (inoculums 0.5 McFarland suspension, 1.5 × 10^8^ CFU/mL) and MIC/MBC (inoculums 5 × 10^5^ CFU/mL) for strains, measured as described in the National Committee for Clinical Laboratory Standards (NCCLS, 1999). Briefly, the growth of bacteria was examined at 37°C in 0.95 mL of BHI broth containing various concentrations of CT. These tubes were inoculated with 5 × 10^5^ colony-forming units (CFU)/mL of an overnight culture grown in BHI broth and incubated at 37°C. After 24 h of incubation, the optical density (OD) was measured spectrophotometrically at 550 nm. Three replicates were measured for each concentration of tested drugs.

### 2.3. Minimum Inhibitory Concentration/Minimum Bactericidal Concentration Assay

The antimicrobial activities of CT against clinical isolates MRSA 16, MSSA 2, VRSA 2, and reference strains were determined* via* the broth dilution method [25]. The minimum inhibitory concentration (MIC) was recorded as the lowest concentration of test samples resulting in the complete inhibition of visible growth. For clinical strains, MIC_50_s and MIC_90_s, defined as MICs at which 50 and 90%, respectively, of the isolates were inhibited, were determined. The minimum bactericidal concentration (MBC) was determined based on the lowest concentration of the extracts required to kill 99.9% of bacteria from the initial inoculum as determined by plating on agar.

### 2.4. Checkerboard Dilution Test

The synergistic combinations were investigated in the preliminary checkerboard method performed using the MRSA, MSSA, and VRSA of clinical isolate strains via MIC and MBC determination [26]. The fractional inhibitory concentration index (FICI) and fractional bactericidal concentration index (FBCI) are the sum of the FICs and FBCs of each of the drugs, which were defined as the MIC and MBC of each drug when used in combination divided by the MIC and MBC of each drug when used alone. The FIC and FBC index was calculated as follows: FIC = (MIC of drug A in combination/MIC of drug A alone) + (MIC of drug B in combination/MIC of drug B alone) and FBC = (MBC of drug A in combination/MBC of drug A alone) + (MBC of drug B in combination/MBC of drug B alone). FIC and FBC indices were interpreted as follows: the FIC index was interpreted as follows: synergy, <0.5; partial synergy, 0.5–0.75; additive effect, 0.76–1.0; indifference, >1.0–4.0; and antagonism, >4.0 [26].

### 2.5. Time-Kill Curves

The bactericidal activities of the drugs evaluated in this study were also evaluated using time-kill curves constructed using the isolated and reference strains. Cultures with an initial cell density of 1–5 × 10^6^ CFU/mL were exposed to the MIC of CT alone or CT (1/2 MIC) plus oxacillin or ampicillin or vancomycin (1/2 MIC). Viable counts were conducted at 0, 0.5, 1, 2, 3, 4, 5, 6, 12, and 24 h by plating aliquots of the samples on agar and subsequent incubation for 24 hours at 37°C. All experiments were repeated several times and colony counts were conducted in duplicate, after which the means were determined.

## 3. Results and Discussion

Many researchers are studying natural products that could be used as antibiotics against MRSA and are employing novel dosing regimens and antimicrobials that would be advantageous for combating the therapeutic problems associated with* S. aureus *[10, 13, 14, 16, 27]. The main bioactive constituents of* S. miltiorrhiza *include water-soluble phenolic acids and lipophilic diterpenoid tanshinones [28–30]. Cryptotanshinone was isolated from dried* S. miltiorrhiza *roots and identified via comparison of their spectral data with the data reported in the literature [23, 24]. ^l^H-NMR (CDCl_3_) *δ* 7.64 (1H, d,* J* = 8.0 Hz), 7.48 (1H, d,* J* = 8.0 Hz), 4.86 (1H, t,* J* = 9.2 Hz), 4.36 (1H, dd,* J* = 6.0 and 6.0 Hz), 3.60 (1H, m), 3.21 (2H, br t), 1.69 (4H, m), 1.36 (3H, d,* J* = 6.4 Hz), 1.31 (6H, s). ^13^C-NMR (CDCl_3_) *δ* 29.67 (C-1), 19.08 (C-2), 37.82 (C-3), 34.86 (C-4), 143.70 (C-5), 132.56 (C-6), 122.50 (C-7), 128.42 (C-8), 126.27 (C-9), 152.37 (C-10), 184.27 (C-11), 175.72 (C-12), 118.30 (C-13), 170.75 (C-14), 81.46 (C-15), 34.62 (C-16), 18.85 (C-17), 31.94 (C-18), and 31.89 (C-19). Among the lipophilic diterpenoid tanshinones, cryptotanshinone, dihydrotanshinone I, tanshinone IIA, and tanshinone I exhibited strong antimicrobial activity against (*Agrobacterium tumefaciens *ATCC 11158,* Escherichia coli *ATCC 29425,* Pseudomonas lachrymans *ATCC 11921,* Ralstonia solanacearum *ATCC 11696, and* Xanthomonas vesicatoria *ATCC 11633) and three Gram-positive bacteria (*Bacillus subtilis *ATCC 11562,* Staphylococcus aureus *ATCC 6538, and* Staphylococcus haemolyticus *ATCC 29970) [18]. Our results of the antibacterial activity showed that the CT exhibited inhibitory activities against isolates MSSA, MRSA, VRSA, and reference stains. The MICs and MBCs values of CT and antibiotics, ampicillin, oxacillin, and vancomycin against MSSA ATCC 25923 and MRSA ATCC 33591 and isolates MSSA 1-2, MRSA 1–16, and VRSA 1-2 are shown in Table 1. The MICs and MBCs values of CT against isolates MSSA 1-2 were in the range of 16 and 32 *μ*g/mL and 64 and 128 *μ*g/mL, isolates MRSA 1–16 in the range of 4–64 *μ*g/mL and 8–128 *μ*g/mL, isolates VRSA 1-2 in the range of 2 and 4 *μ*g/mL and 4 and 16 *μ*g/mL, and reference stains in range of 4 and 64 *μ*g/mL and 16 and 256 *μ*g/mL, respectively. The MICs/MBCs for ampicillin were determined to be either 8/16 or 1024/2048 *μ*g/mL; for oxacillin, either 0.25/1 or 512/2048 *μ*g/mL; for vancomycin, either 0.5/2 or 32/64 *μ*g/mL against reference strains and MSSA 1-2, MRSA 1–16, and VRSA 1-2 isolates. The MIC_50_ and MIC_90_ values of CT for reference strains were 0.5 and 4 *μ*g/mL and 4 and 64 *μ*g/mL, while for MSSA 1-2 and VRSA 1-2 isolates were 0.5–8 *μ*g/mL and 2–32 *μ*g/mL, and for MRSA 1–16 isolates were 0.5–8 *μ*g/mL and 4–64 *μ*g/mL, respectively (Table 1).

Evaluation of* in vivo* effectiveness of the antimicrobial combinations is necessary to generate data that can be extrapolated to the clinical situation as well as predicting relevant concentration of optimal dosing regimens for both agents of the combinations [31, 32]. Combination antibiotic therapy has been studied to promote the effective use of antibiotics in increasing* in vivo *activity of antibiotics, in preventing the spread of drug-resistant strains, and in minimizing toxicity [32, 33]. The combination of oxacillin and CT resulted in a reduction in the MICs/MBCs for isolates VRSA 1-2 and MSSA 1-2, with the MICs/MBCs of 2/16 or 8/128 *μ*g/mL, for oxacillin becoming 0.125/0.125–0.25 *μ*g/mL and reduced by ≥4-fold, evidencing a synergistic effect as defined by a FICI and FBCI of ≤0.5 (Table 2). The combination of oxacillin and CT resulted in a reduction against isolates MRSA 1–16, with the MICs/MBCs values of 0.5–16/2–32 *μ*g/mL, for oxacillin becoming 1–128/4–256 *μ*g/mL and reduced by ≥4-fold, evidencing a synergistic effect as defined by a FICI and FBCI of ≤0.5 except MRSA 4 and 16 of additive (FICI ≥ 0.5) and MRSA 1, 7, and 15 of additive (FBCI ≥ 0.5). The combination of ampicillin and CT resulted in a reduction in the MICs/MBCs for isolates VRSA 1-2 and MSSA 1-2, with the MICs/MBCs of 0.5/1 or 2/4 *μ*g/mL and 4/8 or 8/16, for ampicillin becoming 32/128 or 64/256 *μ*g/mL and 16/64 or 32/128 *μ*g/mL and reduced by ≥4-fold, evidencing a synergistic effect as defined by a FICI and FBCI of ≤0.5 and in most of MRSA testedwere reduced by ≥4-fold evidencing a synergistic effect as defined by a FICI and FBCI of ≤0.5 except MRSA 6, 12, and 15 (FICI ≥ 0.5) and MRSA 6, 11, and 13 (FBCI ≥ 0.5), respectively (Table 3). The combination of vancomycin and CT resulted in a reduction against isolates MRSA 1–16, with the MICs/MBCs values of 1–16/2–32 *μ*g/mL, for vancomycin becoming 0.125–0.5/0.5–2 *μ*g/mL and reduced by ≥4-fold, evidencing a synergistic effect as defined by a FICI and FBCI of ≤ 0.5 except MRSA 6, 11, and 15 of additive (FICI ≥ 0.5) and MRSA 1 and 9 of additive (FBCI ≥ 0.5) and for isolates VRSA 1-2 and MSSA 1-2, with the MICs/MBCs of 0.5/1 or 1/4 *μ*g/mL and 4/8 or 16/16, for vancomycin becoming 4/8 or 8/16 *μ*g/mL and 0.25/2 or 0.25/1 *μ*g/mL and reduced by ≥4-fold, evidencing a synergistic effect as defined by a FICI and FBCI of ≤0.5 except MSSA 1 and 2 of additive (FBCI ≥ 0.5) (Table 4).

**Figure 1:**
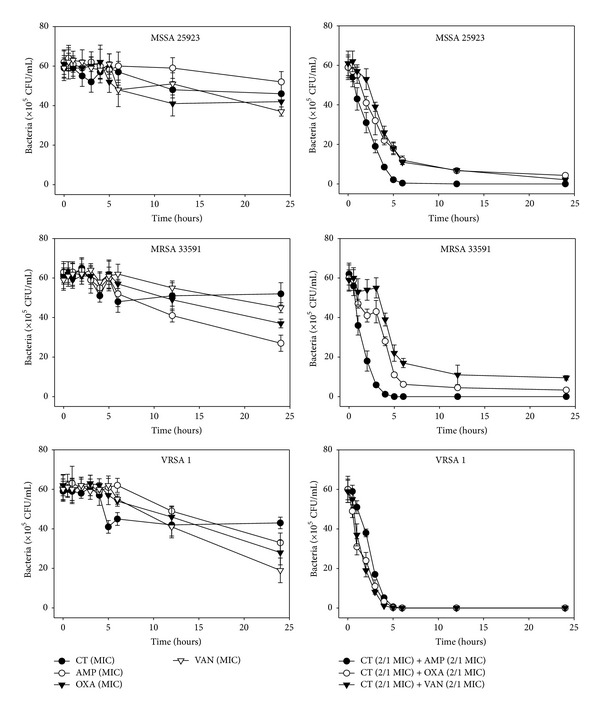
Time-kill curves of MIC or 1/2 MIC of cryptotanshinone (CT), ampicillin (AMP), oxacillin (OXA), and vancomycin (VAN) alone and its combination with MIC_50_ of AMP or OXA, and VAN against VRSA 1 isolates and reference stains, MSSA ATCC 25923 and MRSA ATCC 33591. Bacteria were incubated with MIC of CT (●), AMP, OXA, and VAN, and 1/2 MIC of CT + 1/2 MIC of AMP (○), 1/2 MIC of CT + 1/2 MIC of OXA (*▼*), and 1/2 MIC of CT + 1/2 MIC of VAN (*∇*) over time. CFU: colony-forming units.

**Figure 2:**
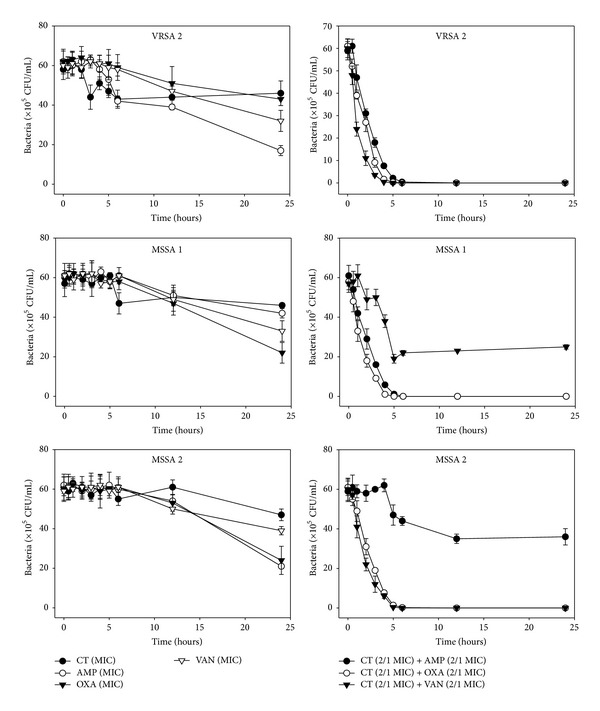
Time-kill curves of MIC or 1/2 MIC of cryptotanshinone (CT), ampicillin (AMP), oxacillin (OXA), and vancomycin (VAN) alone and its combination with 1/2 MIC of AMP or OXA, and VAN against VRSA 2, MSSA 1, and MSSA 2 isolates. Bacteria were incubated with MIC of CT (●), AMP, OXA, and VAN, and 1/2 MIC of CT + 1/2 MIC of AMP (○), 1/2 MIC of CT + 1/2 MIC of OXA (*▼*), and 1/2 MIC of CT + 1/2 MIC of VAN (*∇*) over time. CFU: colony-forming units.

**Figure 3:**
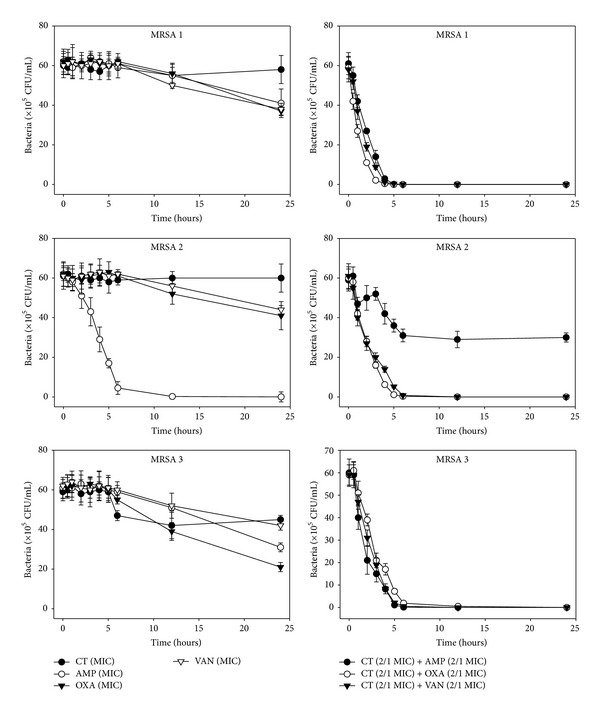
Time-kill curves of MIC or 1/2 MIC of cryptotanshinone (CT), ampicillin (AMP), oxacillin (OXA), and vancomycin (VAN) alone and its combination with 1/2 MIC of AMP or OXA, and VAN against MRSA 1, 2, and 3 isolates. Bacteria were incubated with MIC of CT (●), AMP, OXA, and VAN, and 1/2 MIC of CT + 1/2 MIC of AMP (○), 1/2 MIC of CT + 1/2 MIC of OXA (*▼*), and 1/2 MIC of CT + 1/2 MIC of VAN (*∇*) over time. CFU: colony-forming units.

**Figure 4:**
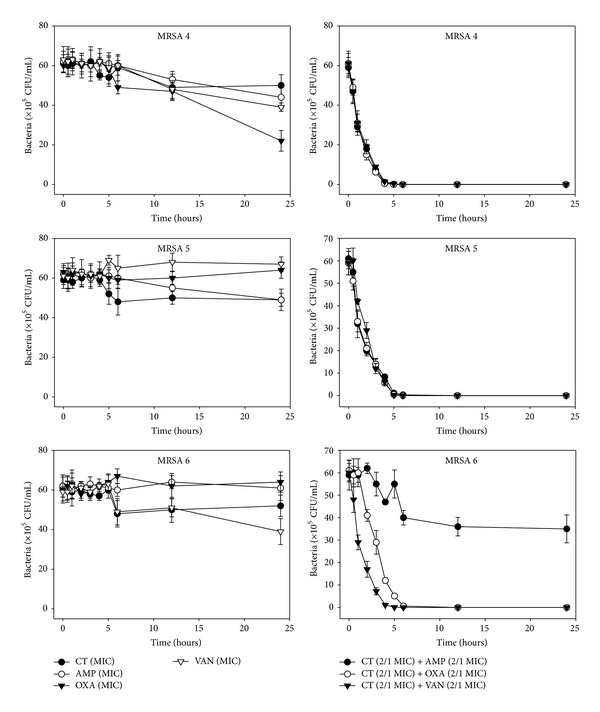
Time-kill curves of MIC or 1/2 MIC of cryptotanshinone (CT), ampicillin (AMP), oxacillin (OXA), and vancomycin (VAN) alone and its combination with 1/2 MIC of AMP or OXA, and VAN against MRSA 4, 5, and 6 isolates. Bacteria were incubated with MIC of CT (●), AMP (○), OXA (*▼*), and VAN (*∇*), and 1/2 MIC of CT + 1/2 MIC of AMP (●), 1/2 MIC of CT + 1/2 MIC of OXA (○), and 1/2 MIC of CT + 1/2 MIC of VAN (*▼*) over time. CFU: colony-forming units.

**Figure 5:**
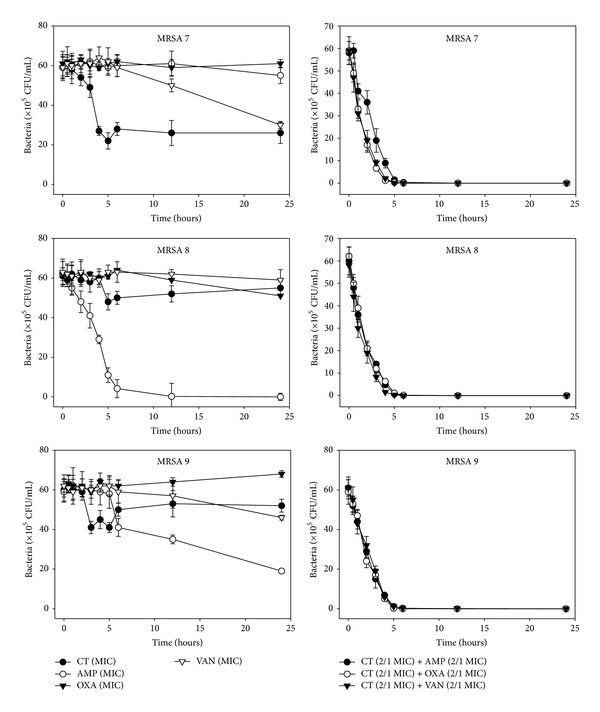
Time-kill curves of MIC or 1/2 MIC of cryptotanshinone (CT), ampicillin (AMP), oxacillin (OXA), and vancomycin (VAN) alone and its combination with 1/2 MIC of AMP or OXA, and VAN against MRSA 7, 8, and 9 isolates. Bacteria were incubated with MIC of CT (●), AMP (○), OXA (*▼*), and VAN (*∇*), and 1/2 MIC of CT + 1/2 MIC of AMP (●), 1/2 MIC of CT + 1/2 MIC of OXA (○), and 1/2 MIC of CT + 1/2 MIC of VAN (*▼*) over time. CFU: colony-forming units.

**Figure 6:**
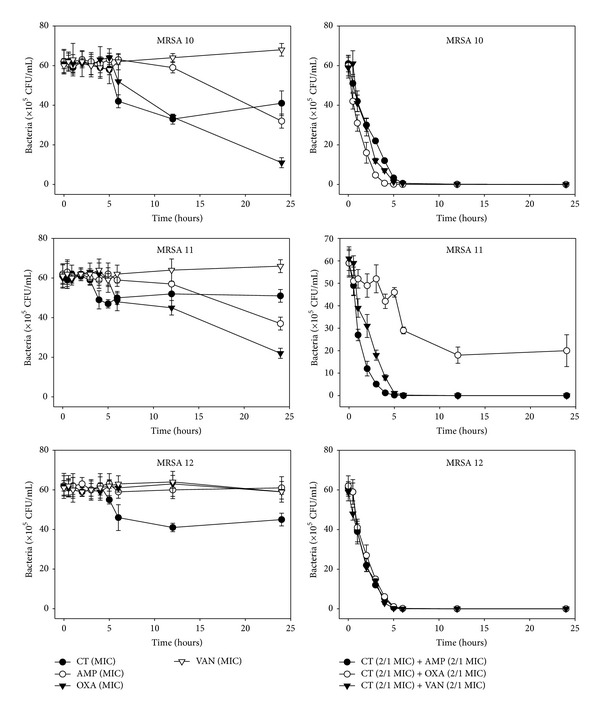
Time-kill curves of MIC or 1/2 MIC of cryptotanshinone (CT), ampicillin (AMP), oxacillin (OXA), and vancomycin (VAN) alone and its combination with 1/2 MIC of AMP or OXA, and VAN against MRSA 10, 11, and 12 isolates. Bacteria were incubated with MIC of CT (●), AMP (○), OXA (*▼*), and VAN (*∇*), and 1/2 MIC of CT + 1/2 MIC of AMP (●), 1/2 MIC of CT + 1/2 MIC of OXA (○), and 1/2 MIC of CT + 1/2 MIC of VAN (*▼*) over time. CFU: colony-forming units.

**Figure 7:**
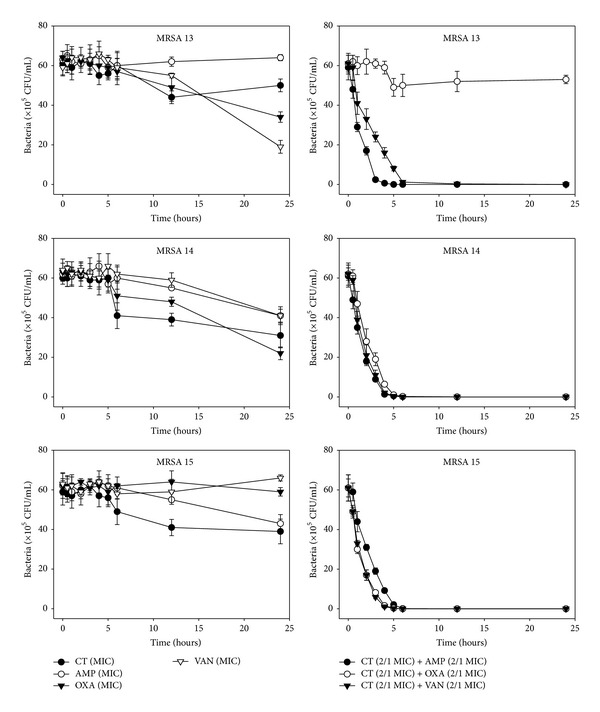
Time-kill curves of MIC or 1/2 MIC of cryptotanshinone (CT), ampicillin (AMP), oxacillin (OXA), and vancomycin (VAN) alone and its combination with 1/2 MIC of AMP or OXA, and VAN against MRSA 13, 14, and 15 isolates. Bacteria were incubated with MIC of CT (●), AMP (○), OXA (*▼*), and VAN (*∇*), and 1/2 MIC of CT + 1/2 MIC of AMP (●), 1/2 MIC of CT + 1/2 MIC of OXA (○), and 1/2 MIC of CT + 1/2 MIC of VAN (*▼*) over time. CFU: colony-forming units.

**Figure 8:**
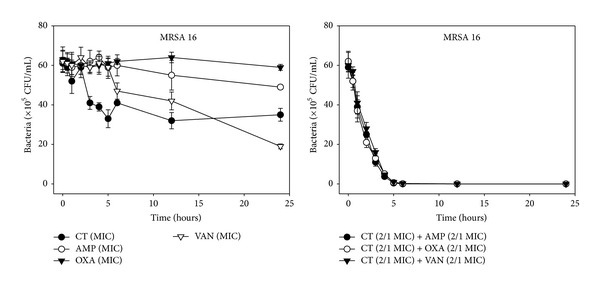
Time-kill curves of MIC or 1/2 MIC of cryptotanshinone (CT), ampicillin (AMP), oxacillin (OXA), and vancomycin (VAN) alone and its combination with 1/2 MIC of AMP or OXA, and VAN against MRSA 16 isolate. Bacteria were incubated with MIC of CT (●), AMP (○), OXA (*▼*), and VAN (*∇*), and 1/2 MIC of CT + 1/2 MIC of AMP (●), 1/2 MIC of CT + 1/2 MIC of OXA (○), and 1/2 MIC of CT + 1/2 MIC of VAN (*▼*) over time. CFU: colony-forming units.

The effects of CT administered in combination with oxacillin and/or ampicillin and/or vancomycin against standard (MSSA and MRSA) and clinical isolates of MSSA (1, 2), VRSA (1,2), and MRSA (MRSA 1–16) were confirmed by time-kill curve experiments (Figures 1, 2, 3, 4, 5, 6, 7, and 8). Cultures of each strain of bacteria with a cell density of 10^6^ CFU/mL were exposed to the MIC of CT and antibiotics alone or CT (1/2 MIC) with oxacillin (1/2 MIC), ampicillin (1/2 MIC), or vancomycin (1/2 MIC). We observed that 30 minutes of CT treatment with ampicillin, oxacillin, or vancomycin resulted in an increased rate of killing as compared to that observed with CT (MIC) alone. A profound bactericidal effect was exerted when a combination of drugs was utilized. The growth of the tested bacteria was completely attenuated after 2–5 h of treatment with the 1/2 MIC of CT, regardless of whether it was administered alone or with oxacillin (1/2 MIC), ampicillin (1/2 MIC), or vancomycin (1/2 MIC) (Figures 1–8).

It has been reported that some plant-derived compounds can improve the* in vitro* activity of some cell-wall inhibiting antibiotics by directly attacking the same target site, that is, peptidoglycan [7, 9, 34]. CT exhibits antimicrobial activity against a broad range of Gram-positive bacteria, including* S. aureus*, and Gram-negative bacteria as well as other microorganisms [21, 22]. Despite the pharmacological activities, potential risks regarding combination use of* S. miltiorrhiza* and drugs have been observed [28, 35, 36]. Scientific findings concluded* S. miltiorrhiza *fraction, and its components (cryptotanshinone and dihydrotanshinone I) showed antibacterial activity against a broad range of bacteria, including the broad range of Gram-positive bacteria and Gram-negative bacteria, and superoxide radicals are considered important in the antibacterial actions of the agents [18]. The Gram-positive bacteria-specific properties of CT are caused by the inhibition of RNA and protein synthesis, rather than by attacking the bacterial membrane [21, 22].

In conclusion, CT of* S. miltiorrhiza *is expected to be recognized as natural sources for the development of new functional drugs against multiresistant* S. aureus*, MRSA and VRSA.

## Figures and Tables

**Table 1:** Antibacterial activity of cryptotanshinone and antibiotics in isolated MRSA, VRSA, MSSA, and some of reference bacteria.

Samples	Cryptotanshinone (*μ*g/mL)			Ampicillin	Oxacillin	Vancomycin
	MIC_50<_	MIC_90<_	MIC/MBC	MIC/MBC (*μ*g/mL)		
MSSA ATCC 25923^1^	4	64	64/256	8/16	0.25/1	0.5/2
MRSA ATCC 33591^2^	0.5	4	4/16	512/2048	8/16	1/4
VRSA 3A063^3^	0.5	2	2/4	512/2048	512/1024	16/32
VRSA 3A066^4^	1	4	4/16	128/256	512/1024	32/64
MSSA 1^5^	2	16	16/64	1024/2048	0.5/1	1/4
MSSA 2^6^	8	32	32/128	256/512	1/2	1/2
MRSA 1	2	16	16/32	128/256	128/256	1/2
MRSA 2	8	64	64/128	256/256	8/32	2/4
MRSA 3	1	4	4/16	128/512	128/512	2/4
MRSA 4	0.5	4	4/8	128/256	512/2048	1/2
MRSA 5	2	64	64/128	128/512	512/1024	1/2
MRSA 6	0.5	4	4/16	64/128	64/256	1/2
MRSA 7	2	8	8/16	128/256	512/1024	1/4
MRSA 8	4	32	32/64	256/256	512/2048	1/4
MRSA 9	2	8	8/16	64/128	512/1024	1/2
MRSA 10	2	16	16/64	128/256	512/1024	1/2
MRSA 11	4	8	8/32	64/128	64/128	1/2
MRSA 12	1	4	4/8	128/256	128/256	1/4
MRSA 13	4	32	32/128	128/256	32/128	1/2
MRSA 14	0.5	8	8/16	64/128	8/16	1/4
MRSA 15	0.5	4	4/16	64/128	128/256	1/4
MRSA 16	2	8	8/16	128/512	128/512	1/2

^1^MSSA (ATCC 25923): reference strain methicillin-sensitive *Staphylococcus aureus.*

^2^MRSA (ATCC 33591): reference strain methicillin-resistant *Staphylococcus aureus.*

^3^VRSA 3A063: vancomycin-resistant *Staphylococcus aureus* isolated clinically.

^4^VRSA 3A066: vancomycin-resistant *Staphylococcus aureus* isolated clinically.

^5^MSSA (1-2): methicillin-sensitive *Staphylococcus aureus* isolated clinically.

^6^MRSA (1–16): methicillin-resistant *Staphylococcus aureus *isolated clinically.

**Table 2:** Synergistic effects of the cryptotanshinone with oxacillin in isolated MRSA, VRSA, MSSA, and some of reference bacteria.

Samples	Agent	MIC/MBC (*μ*g/mL)		FIC/FBC	FICI/FBCI^2^	Outcome
		Alone	Combination^1^			
MSSA ATCC 25923^3^	Cryptotanshinone	64/256	8/32	0.125/0.125	0.375/0.375	**Synergistic/synergistic**
	Oxacillin	0.25/1	0.0625/0.25	0.25/0.25		
MRSA ATCC 33591^4^	Cryptotanshinone	4/16	1/4	0.25/0.25	0.5/0.5	**Synergistic/synergistic**
	Oxacillin	8/16	2/4	0.25/0.25		
VRSA 3A063^5^	Cryptotanshinone	2/4	0.5/2	0.25/0.5	0.375/0.375	**Synergistic/synergistic**
	Oxacillin	512/1024	64/128	0.125/0.125		
VRSA 3A066^6^	Cryptotanshinone	4/16	1/4	0.25/0.25	0.375/0.5	**Synergistic/synergistic**
	Oxacillin	512/1024	64/256	0.125/0.25		
MSSA 1^7^	Cryptotanshinone	16/64	2/8	0.125/0.125	0.375/0.25	**Synergistic/synergistic **
	Oxacillin	0.5/1	0.125/0.125	0.25/0.125		
MSSA 2	Cryptotanshinone	32/128	16/32	0.5/0.25	0.375/0.375	**Synergistic/synergistic**
	Oxacillin	1/2	0.125/0.25	0.125/0.125		
MRSA 1^8^	Cryptotanshinone	16/32	4/16	0.25/0.5	0.5/0.75	**Synergistic/additive**
	Oxacillin	128/256	32/64	0.25/0.25		
MRSA 2	Cryptotanshinone	64/128	16/16	0.25/0.125	0.5/0.375	**Synergistic/synergistic**
	Oxacillin	8/32	2/8	0.25/0.25		
MRSA 3	Cryptotanshinone	4/16	0.5/4	0.125/0.25	0.375/0.375	**Synergistic/synergistic**
	Oxacillin	128/512	32/64	0.25/0.125		
MRSA 4	Cryptotanshinone	4/8	2/2	0.5/0.25	0.75/0.375	**Additive/synergistic**
	Oxacillin	512/2048	128/256	0.25/0.125		
MRSA 5	Cryptotanshinone	64/128	16/32	0.25/0.25	0.375/0.5	**Synergistic/synergistic**
	Oxacillin	512/1024	64/256	0.125/0.25		
MRSA 6	Cryptotanshinone	4/16	1/4	0.25/0.25	0.5/0.5	**Synergistic/synergistic**
	Oxacillin	64/256	16/64	0.25/0.25		
MRSA 7	Cryptotanshinone	8/16	2/8	0.25/0.5	0.375/0.75	**Synergistic/additive**
	Oxacillin	512/1024	64/256	0.125/0.25		
MRSA 8	Cryptotanshinone	32/64	8/32	0.25/0.5	0.5/0.625	**Synergistic/synergistic**
	Oxacillin	512/2048	128/256	0.25/0.125		
MRSA 9	Cryptotanshinone	8/16	2/4	0.25/0.25	0.375/0.3125	**Synergistic/synergistic**
	Oxacillin	512/1024	64/64	0.125/0.0625		
MRSA 10	Cryptotanshinone	16/64	2/8	0.125/0.125	0.3125/0.25	**Synergistic/synergistic**
	Oxacillin	512/1024	32/128	0.0625/0.125		
MRSA 11	Cryptotanshinone	8/32	1/4	0.125/0.125	0.375/0.375	**Synergistic/synergistic**
	Oxacillin	64/128	16/64	0.25/0.5		
MRSA 12	Cryptotanshinone	4/8	1/2	0.25/0.25	0.5/0.5	**Synergistic/synergistic**
	Oxacillin	128/256	32/64	0.25/0.25		
MRSA 13	Cryptotanshinone	32/128	8/32	0.25/0.25	0.5/0.375	**Synergistic/synergistic**
	Oxacillin	32/128	8/8	0.25/0.125		
MRSA 14	Cryptotanshinone	8/16	2/4	0.25/0.25	0.375/0.5	**Synergistic/synergistic**
	Oxacillin	8/16	1/4	0.125/0.25		
MRSA 15	Cryptotanshinone	4/16	1/8	0.25/0.5	0.5/0.75	**Synergistic/additive**
	Oxacillin	128/256	32/64	0.25/0.25		
MRSA 16	Cryptotanshinone	8/16	2/4	0.25/0.25	0.75/0.375	**Additive/synergistic**
	Oxacillin	128/512	64/64	0.5/0.125		

^1^The MIC and MBC of cryptotanshinone with oxacillin.

^2^The FIC index.

^3^MSSA (ATCC 25923): reference strain methicillin-sensitive *Staphylococcus aureus.*

^4^MRSA (ATCC 33591): reference strain methicillin-resistant *Staphylococcus aureus.*

^5^VRSA 3A063: vancomycin-resistant *Staphylococcus aureus* isolated clinically.

^6^VRSA 3A066: vancomycin-resistant *Staphylococcus aureus* isolated clinically.

^7^MSSA (1-2): methicillin-sensitive *Staphylococcus aureus* isolated clinically.

^8^MRSA (1–16): methicillin-resistant *Staphylococcus aureus* isolated clinically.

**Table 3:** Synergistic effects of cryptotanshinone with ampicillin in isolated MRSA, VRSA, MSSA, and some of reference bacteria.

Samples	Agent	MIC/MBC (*μ*g/mL)		FIC/FBC	FICI/FBCI^2^	Outcome
		Alone	Combination^1^			
MSSA ATCC 25923^3^	Cryptotanshinone	64/256	16/64	0.25/0.25	0.5/0.75	**Synergistic/additive**
	Ampicillin	8/16	2/8	0.25/0.5		
MRSA ATCC 33591^4^	Cryptotanshinone	4/16	1/4	0.25/0.25	0.5/0.5	**Synergistic/synergistic**
	Ampicillin	512/2048	128/512	0.25/0.25		
VRSA 3A063^5^	Cryptotanshinone	2/4	0.5/2	0.25/0.5	0.5/0.375	**Synergistic/synergistic **
	Ampicillin	512/2048	128/256	0.25/0.125		
VRSA 3A066^6^	Cryptotanshinone	4/16	1/4	0.25/0.25	0.5/0.5	**Synergistic/synergistic**
	Ampicillin	128/256	32/64	0.25/0.25		
MSSA 1^7^	Cryptotanshinone	16/64	4/8	0.25/0.125	0.5/0.375	**Synergistic/synergistic**
	Ampicillin	64/128	16/32	0.25/0.25		
MSSA 2	Cryptotanshinone	32/128	8/16	0.25/0.125	0.5/0.375	**Synergistic/synergistic**
	Ampicillin	256/512	64/128	0.25/0.25		
MRSA 1^8^	Cryptotanshinone	16/32	4/8	0.25/0.25	0.5/0.5	**Synergistic/synergistic**
	Ampicillin	128/256	32/64	0.25/0.25		
MRSA 2	Cryptotanshinone	64/128	16/32	0.25/0.25	0.5/0.5	**Synergistic/synergistic**
	Ampicillin	64/256	16/64	0.25/0.25		
MRSA 3	Cryptotanshinone	4/16	1/2	0.25/0.125	0.5/0.25	**Synergistic/synergistic**
	Ampicillin	128/512	32/64	0.25/0.125		
MRSA 4	Cryptotanshinone	4/8	1/2	0.25/0.25	0.5/0.5	**Synergistic/synergistic**
	Ampicillin	128/256	32/64	0.25/0.25		
MRSA 5	Cryptotanshinone	64/128	16/32	0.25/0.25	0.5/0.5	**Synergistic/synergistic**
	Ampicillin	256/512	64/128	0.25/0.25		
MRSA 6	Cryptotanshinone	4/16	1/2	0.25/0.125	0.75/0.625	**Additive/additive**
	Ampicillin	64/128	32/64	0.5/0.5		
MRSA 7	Cryptotanshinone	8/16	2/4	0.25/0.25	0.5/0.5	**Synergistic/synergistic**
	Ampicillin	128/256	32/64	0.25/0.25		
MRSA 8	Cryptotanshinone	32/64	8/16	0.25/0.25	0.5/0.375	**Synergistic/synergistic**
	Ampicillin	32/64	8/8	0.25/0.125		
MRSA 9	Cryptotanshinone	8/16	2/4	0.25/0.25	0.5/0.5	**Synergistic/synergistic**
	Ampicillin	64/128	16/32	0.25/0.25		
MRSA 10	Cryptotanshinone	16/64	2/8	0.125/0.125	0.375/0.375	**Synergistic/synergistic**
	Ampicillin	128/256	32/64	0.25/0.25		
MRSA 11	Cryptotanshinone	8/32	2/8	0.25/0.25	0.5/0.75	**Synergistic/additive**
	Ampicillin	64/128	16/64	0.25/0.5		
MRSA 12	Cryptotanshinone	4/8	1/2	0.25/0.25	0.75/0.5	**Additive/synergistic**
	Ampicillin	128/256	64/64	0.5/0.25		
MRSA 13	Cryptotanshinone	32/128	8/32	0.25/0.25	0.5/0.75	**Synergistic/additive**
	Ampicillin	128/256	32/128	0.25/0.5		
MRSA 14	Cryptotanshinone	8/16	2/4	0.25/0.25	0.375/0.5	**Synergistic/synergistic**
	Ampicillin	64/128	8/32	0.125/0.25		
MRSA 15	Cryptotanshinone	4/16	2/4	0.5/0.25	0.75/0.5	**Additive/synergistic**
	Ampicillin	64/128	16/32	0.25/0.25		
MRSA 16	Cryptotanshinone	8/16	2/4	0.25/0.25	0.5/0.375	**Synergistic/synergistic**
	Ampicillin	128/512	32/64	0.25/0.125		

^1^The MIC and MBC of cryptotanshinone with ampicillin.

^2^The FIC index.

^3^MSSA (ATCC 25923): reference strain methicillin-sensitive *Staphylococcus aureus.*

^4^MRSA (ATCC 33591): reference strain methicillin-resistant *Staphylococcus aureus.*

^5^VRSA 3A063: vancomycin-resistant *Staphylococcus aureus* isolated clinically.

^6^VRSA 3A066: vancomycin-resistant *Staphylococcus aureus* isolated clinically.

^7^MSSA (1-2): methicillin-sensitive *Staphylococcus aureus* isolated clinically.

^8^MRSA (1–16): methicillin-resistant *Staphylococcus aureus *isolated clinically.

**Table 4:** Synergistic effects of cryptotanshinone with vancomycin in isolated MRSA, VRSA, MSSA, and some of reference bacteria.

Samples	Agent	MIC/MBC (*μ*g/mL)		FIC/FBC	FICI/FBCI^2^	Outcome
		Alone	Combination^1^			
MSSA ATCC 25923^3^	Cryptotanshinone	64/256	16/64	0.25/0.25	0.5/0.5	**Synergistic/synergistic**
	Vancomycin	0.5/2	0.125/0.5	0.25/0.25		
MRSA ATCC 33591^4^	Cryptotanshinone	4/16	1/4	0.25/0.25	0.5/0.5	**Synergistic/synergistic**
	Vancomycin	1/4	0.25/1	0.25/0.25		
VRSA 3A063^5^	Cryptotanshinone	2/4	0.5/1	0.125/0.25	0.375/0.5	**Synergistic/synergistic**
	Vancomycin	16/32	4/8	0.25/0.25		
VRSA 3A066^6^	Cryptotanshinone	4/16	1/4	0.25/0.25	0.5/0.5	**Synergistic/synergistic**
	Vancomycin	32/64	8/16	0.25/0.25		
MSSA 1^7^	Cryptotanshinone	16/64	4/16	0.25/0.25	0.5/0.75	**Synergistic/additive **
	Vancomycin	1/4	0.25/2	0.25/0.5		
MSSA 2	Cryptotanshinone	32/128	8/16	0.25/0.125	0.5/0.625	**Synergistic/additive**
	Vancomycin	1/2	0.25/1	0.25/0.5		
MRSA 1^8^	Cryptotanshinone	16/32	4/8	0.25/0.25	0.5/0.5	**Synergistic/synergistic**
	Vancomycin	1/2	0.25/0.5	0.25/0.25		
MRSA 2	Cryptotanshinone	64/128	16/32	0.25/0.25	0.5/0.75	**Synergistic/additive **
	Vancomycin	2/4	0.5/2	0.25/0.5		
MRSA 3	Cryptotanshinone	4/16	1/4	0.25/0.25	0.375/0.5	**Synergistic/synergistic**
	Vancomycin	2/4	0.25/1	0.125/0.25		
MRSA 4	Cryptotanshinone	4/8	1/2	0.25/0.25	0.5/0.5	**Synergistic/synergistic**
	Vancomycin	1/2	0.25/0.5	0.25/0.25		
MRSA 5	Cryptotanshinone	64/128	16/32	0.25/0.25	0.5/0.5	**Synergistic/synergistic**
	Vancomycin	1/2	0.25/0.5	0.25/0.25		
MRSA 6	Cryptotanshinone	4/16	2/4	0.5/0.25	0.75/0.5	**Additive/synergistic**
	Vancomycin	1/2	0.25/0.5	0.25/0.25		
MRSA 7	Cryptotanshinone	8/16	2/4	0.25/0.25	0.5/0.375	**Synergistic/synergistic**
	Vancomycin	1/4	0.25/0.5	0.25/0.125		
MRSA 8	Cryptotanshinone	32/64	8/16	0.25/0.25	0.5/0.375	**Synergistic/synergistic**
	Vancomycin	1/4	0.25/0.5	0.25/0.125		
MRSA 9	Cryptotanshinone	8/16	2/4	0.25/0.25	0.5/0.5	**Synergistic/synergistic**
	Vancomycin	1/2	0.25/0.5	0.25/0.25		
MRSA 10	Cryptotanshinone	16/64	4/8	0.25/0.25	0.5/0.75	**Synergistic/additive**
	Vancomycin	1/2	0.25/1	0.25/0.5		
MRSA 11	Cryptotanshinone	8/32	2/8	0.25/0.25	0.75/0.5	**Additive/synergistic**
	Vancomycin	1/2	0.5/0.5	0.5/0.25		
MRSA 12	Cryptotanshinone	4/8	1/2	0.25/0.25	0.5/0.375	**Synergistic/synergistic**
	Vancomycin	1/4	0.25/0.5	0.25/0.125		
MRSA 13	Cryptotanshinone	32/128	16/32	0.25/0.25	0.5/0.5	**Synergistic/synergistic**
	Vancomycin	1/2	0.25/0.5	0.25/0.25		
MRSA 14	Cryptotanshinone	8/16	2/4	0.25/0.25	0.5/0.5	**Synergistic/synergistic**
	Vancomycin	1/4	0.25/1	0.25/0.25		
MRSA 15	Cryptotanshinone	4/16	2/4	0.5/0.25	0.75/0.375	**Additive/synergistic**
	Vancomycin	1/4	0.25/0.5	0.25/0.125		
MRSA 16	Cryptotanshinone	8/16	2/4	0.25/0.25	0.375/0.5	**Synergistic/synergistic**
	Vancomycin	1/2	0.125/0.5	0.125/0.25		

^1^The MIC and MBC of cryptotanshinone with vancomycin.

^2 ^The FIC index.

^3^MSSA (ATCC 25923): reference strain methicillin-sensitive *Staphylococcus aureus.*

^4^MRSA (ATCC 33591): reference strain methicillin-resistant *Staphylococcus aureus.*

^5^VRSA 3A063: vancomycin-resistant *Staphylococcus aureus* isolated clinically.

^6^VRSA 3A066: vancomycin-resistant *Staphylococcus aureus* isolated clinically.

^7^MSSA (1-2): methicillin-sensitive *Staphylococcus aureus* isolated clinically.

^8^MRSA (1–16): methicillin-resistant *Staphylococcus aureus *isolated clinically.
